# On the Cure of Whitlow by Compression

**Published:** 1818-03

**Authors:** William Balfour

**Affiliations:** Edinburgh.


*For the London Medical and Physical Journal.*

### *On the Cure of Whitlow by Compression;* by WILLIAM BALFOUR, M.D. of Edinburgh.

TWO modes of curing whitlow have prevailed, the very opposite of each other: the one tends to promote, the other to prevent, the formation of matter. Such diversity of practice must have originated, one would think, in discrepancy of opinion, with regard to the disease being disposed to run a certain course. No man, surely, who thinks that the formation of matter can, with propriety, be prevented, would advise emollients; none, on the other hand, would prescribe astringents, who believed the formation of matter necessary to the cure of the disease.

The emollient plan of treating whitlow is now, I believe, generally laid aside; and, though the practice of applying astringents rests on sound principles, it is not efficient. It is but now and then, and in slight cases, that astringents will arrest the progress of the disease. If distention was confined to superficial vessels, as in slight cases of sore throat, astringents would quickly prevent the formation of matter in most instances. But, as the complaint is often deep-seated, it is impossible that the most powerful astringents we possess can have much effect in diminishing the calibre, or in strengthening the tone of the distended vessels.

But it is not very evident, that the principle on which astringents produce their beneficial effects in whitlow has had its due consideration. If it had, we surely should not hear of some, whom we are bound to consider among the best informed of the profession, applying them warm. Heat must increase the distension of vessels already over distended, and produce that effusion which it is the object of the practitioner to prevent.

My success in curing and relieving rheumatic complaints, by merely unloading and supporting the vessels of the parts affected, as detailed in my treatise on that subject, suggested to me the practicability of obviating and removing other inflammatory affections by the same simple means. This practice, therefore, I have adopted in whitlow, and with all the success that could be desired. A careful observation, indeed, of the phenomena exhibited in the first stage of whitlow, (and to this stage alone are my observations confined,) naturally leads to compression as the most likely means of arresting the disease. When the pain of incipient whitlow first excites a patient's attention, he perceives, in the part affected, a degree of tension which he did not observe before. An accurate observer must, therefore, connect, in his mind, this tension and pain, as cause and effect. In this he must be confirmed, on observing, that, with tension, pain also increases. We know, indeed, from experience, that tension is the cause of pain: for, no sooner are the vessels of the affected part unloaded, by leeches or the scalpel, than pain abates in proportion. It is evident, therefore, that whatever is capable of restoring the balance between the arteries and veins, must arrest the progress of the disease. This compression can do, as illustrated in the following cases.

Case 1.—About two years ago, Janet Bain, aged 35, a servant, was seized with whitlow in one of her thumbs, after having been employed a whole day in the bleaching-green during very cold weather. When she applied to me, the whole thumb was swelled, painful, and of a livid colour; she knew no cause for her complaint. I never saw any case of whitlow of so deep a colour, and apprehended the very worst issue. I applied compression with the hand for a considerable time, and then with a bandage. I had little or no hope, however, of these means proving of much advantage. From the pain and appearance of the thumb, I was afraid that mortification would quickly supervene. The patient did not call upon me again for some days, when I found her thumb perfectly well. The family surgeon had called soon after she left me, and she was ordered to put herself under his care. The bandage I applied being still about the thumb, she satisfied herself with a verbal account of the pain and appearance, without informing him she had consulted any one. The surgeon advised a warm-vinegar poultice. With this, the patient would not comply; but agreed to adopt so much of his advice only, as she imagined was not inconsistent with my directions. She, therefore, dipped her thumb in cold vinegar, without undoing the bandage. This had all the effect of tightening the bandage, without undoing it; and the cure was thus speedily completed.

Case 2.—Towards the latter end of 1816, a lady received a severe blow on the middle joint of her middle finger, right hand, from the falling of a window upon it, the pulley of which was broken. The pain, which was extreme, soon abated, but was followed by swelling of the whole finger, extending over a considerable portion of the back of the hand, especially in the course of the extensor tendon of the finger. A fortnight elapsed, from the evening of the accident, before I was consulted. During this period, the tension and pain had gradually increased, and were now extreme. The whole of the finger and back of the hand were pained, but chiefly the middle joint and first phalanx; and some idea may be formed of the distress of the patient, from the circumstance of my being called to her, for the first time, at eleven o'clock at night. The tension, throbbing, and pain, were insufferable, and must soon have terminated in suppuration. I applied compression over all the parts affected, with my fingers and thumb; at first, in a very gentle manner, and gradually with more freedom, as the feelings of the patient would permit. By these means, I reduced the swelling perceptibly in a short time, and then applied a narrow fillet circularly the whole length of the finger, and with a degree of tightness which could not have been suffered without the previous handling, and which was still regulated by the patient's feelings. I directed her to keep the joints of the fingers a little elevated during the night, and to slacken the bandage if necessary. Next day, I found she passed the night comfortably, without having occasion to undo the bandage; and that both swelling and pain had greatly abated. All the symptoms, indeed, were so much ameliorated, that the patient insisted on omitting the bandage. On the following day, the second from the time I was called, she was menaced with a return of pain. I was again sent for, and renewed my operations. In a day or two the pain ceased entirely and permanently.

Case 3.—About twelve months ago, a young lady was seized with whitlow in one of her thumbs, without any assignable cause. After suffering a gradual increase of pain for ten or twelve days, she became, at last, quite impatient and consulted me. I found the whole of the last phalanx tense, red, shining, and excruciatingly painful, especially about the root of the nail. The whole hand was, in some degree, affected; and the pain shot as high as the elbow-joint. On examination, I told her, suppuration had either taken place, or was on the point of it; so that she was rather late in applying, for my mode of treatment to take effect. I applied compression, however, with the hand, in a degree she could easily bear, with the view of preventing extensive suppuration, and then a narrow fillet. This operation was repeated three or four times in the course of two days, when pain and swelling disappeared, leaving a single speck of pus at the point of the thumb, immediately under the skin;—to this, I gave vent by the slightest touch of a lancet, and the wound healed up immediately. That the formation of pus was checked by these operations, after it was begun, there cannot, I think, be a doubt.

Case 4.—Colin Mackenzie, aged 46, applied to me for advice, on the 6th of August, 1817, on account of the little finger of his right hand, which was swelled and excruciatingly painful, from the middle joint to the point inclusive. He knew no cause for the complaint, which had been gradually increasing for eight days, and now prevented him entirely from following his occupation. These symptoms, and the glossy appearance of the finger, left no room to doubt that this was a case of severe whitlow. I applied compression over all the parts affected, with my finger and thumb, moved from place to place in succession. The joints, indeed, were so very painful, that the patient could scarcely suffer them to be touched. But, proceeding leisurely and with caution, I overcame this tenderness in about fifteen minutes. I then bound up the finger its whole length, with a piece of narrow tape,—regulating the degree of compression by the patient's feelings. This took place at eight o'clock in the evening, and I directed him to slacken the bandage before going to bed, if he found it too tight. This he did, and also next day, while at work. He called on me again at eight o'clock in the evening of the 7th, twenty-four hours after the first operation, when I found the swelling generally gone, and the pain greatly abated, except at the middle joint, which was originally most painful, and to which the patient did not re-apply the bandage sufficiently tight when he had occasion to undo it. This joint was, therefore, more swelled and pained than ever. I applied compression to it as before, till the patient admitted that both tension and pain were relieved; and then the bandage. Next morning, at ten o'clock, the tension and pain were so nearly removed, that almost complete plexion of the finger was restored. Thus, in the short space of thirty-eight hours, was this man relieved from a complaint, which, under the usual treatment, must infallibly have ended in suppuration and protracted suffering.

Case 5.—James Briddet, a tanner, aged 25, applied to me on the 25th of August, with whitlow in one of his thumbs. He knew no cause for the complaint, which had existed a week, and was now likely to prevent him following his occupation. The whole thumb was affected, but particularly the two joints; and, the inflammation about the first joint and ball was such as to make me observe to the patient, that certainly the parts were coloured by the substances he had occasion to handle when at work. He assured me the appearance of the parts was entirely the effect of disease. I found this fellow quite sceptical with regard to my mode of cure. When I had handled the parts and applied a bandage, I desired him to call next day. He looked at me, as if he would have said, "Is this all you are to do for me?" He called next morning, however, when inflammation and swelling were considerably abated, especially about the first joint. The last joint was still painful. In the evening of the same day, this joint was also much better. Next day, the third inclusive, swelling and pain were almost entirely gone; and the patient had the free use of his thumb. I now asked him, if he was not, at first, quite distrustful in the mode of cure I adopted? He admitted he was, expressed his surprise at the result, made his acknowledgments, and went about his business.

Case 6.—Peter Fraser received an injury on the 26th of December last, by having his left thumb bent forcibly backwards, when assisting at lifting a heavy stone. When he applied to me on the 29th, he complained of having passed three days in great agony, and three sleepless nights. The pain was confined to the first joint, but the swelling extended a considerable way upwards. I never handled a more excruciatingly painful case, and I believed it must soon have terminated in suppuration. Such was also the opinion of Dr. Anderson, of New York, who happened to be with me when the patient presented himself. I told that gentleman, that, exquisitely painful as was the complaint, I had no doubt of curing it in a week, without any other application than my own fingers and a piece of narrow tape. The cure was completed in six days, inclusive of that on which the patient applied to me.

I shall make some observations on these cases hereafter; in the mean time, I would say of the mode of cure, "*Si quid novesti rectius istis, candidus imperti; si non, his utere mecum.*"

*January* 8, 1818.

